# A study on factors influencing the national carbon emission trading price in China

**DOI:** 10.1371/journal.pone.0333788

**Published:** 2025-10-07

**Authors:** Mingzhu Liao, Feng Long, Xue Tian, Fenfen Bi, Wei Tian, Xiao Li, Chazhong Ge

**Affiliations:** 1 School of Management, Hangzhou Dianzi University, Hangzhou, China; 2 The Center for Environmental Tax, Chinese Academy of Environmental Planning, Beijing, China; 3 National Joint Research Center for Ecological Conservation and High Quality Development of the Yellow River Basin, Beijing, China; Central Queensland University, AUSTRALIA

## Abstract

On 16 July 2021, China officially launched its national carbon emissions trading market, which has since become the largest market in the world in terms of coverage of greenhouse gas emissions and plays an important role in combating global climate change. This study selects the national carbon emissions trading price data from July 16, 2021 to August 31, 2024, which records the price fluctuation characteristics in the early stage of the market (covering only the stage of the electric power industry), which not only provides a historical reference for the subsequent inclusion of the industry, but also provides an important basis for evaluating the effectiveness of the market construction and the optimization of the policy. At the same time, the VEC model is used to study the dynamic relationship between the national carbon emission trading price and key variables, including energy prices, macroeconomic conditions, the development of the power industry, international carbon prices, carbon emissions from the power industry, and the trading volume of national carbon emission quotas. The results show that there is a long-term equilibrium relationship between the national carbon trading price and the variables, which provides a scientific basis for setting a reasonable fluctuation range of the carbon price. Furthermore, impulse response and variance decomposition analyses were conducted to evaluate the short-term dynamic effects of each variable on the national carbon emission trading price. The results reveal that the trading volume of national carbon emission quotas and the development of the power industry exert significant influence on the national carbon emission trading price. In addition, the carbon emissions of the electric power industry have a positive impact on the national carbon emissions trading price in the short-term situation, and in the long-term situation, they have a negative impact. This provides a parameter calibration basis for the expansion of the industry and a reference for policy making, which is important for the construction of the national carbon emission trading market with international influence. The marginal contributions of this study are:(1) forming a new combination of variables for a comprehensive analysis of the national carbon emissions trading market; (2) the empirical results uncover new insights into the dynamics of the national carbon price; and (3) providing empirical evidence for improving the institutional system of the national carbon emissions trading market.

As global climate issues intensify, extreme meteorological events are becoming more frequent, sea levels continue to rise, and seawater intrusion is increasingly pronounced, leading to heightened international attention to climate and environmental challenges. The extensive release of greenhouse gases (GHGs) is widely recognized as the primary driver of rising global temperatures. To mitigate GHG emissions, the United Nations established emission rights under the 1997 Kyoto Protocol and introduced a carbon emission trading market. This market internalizes the negative externalities of emissions by converting them into costs, serving as a policy tool that leverages market mechanisms to control and reduce GHG emissions [1]. As the top contributor to global carbon emissions, China bears significant responsibility for carbon emission reduction and environmental governance [2]. The national carbon emission trading market (hereinafter referred to as the national carbon market) currently encompasses 2,257 key emission units in the power industry, covering approximately 5.1 billion tons of CO2 emissions annually, which accounts for over 40% of China’s total CO2 emissions. This makes it the largest carbon market globally in terms of GHG emissions coverage [3]. On September 9, 2024, the Ministry of Ecology and Environment released the Work Plan for Including the Cement, Steel, and Electrolyte Aluminum Industries in the National Carbon Emissions Trading Market (Draft for Comments), marking the initial expansion phase of the national carbon market. As the market continues to evolve, establishing a reliable pricing mechanism is critical for its effective operation. However, the national carbon emission trading price (hereinafter referred to as the national carbon price) exhibits significant volatility and is influenced by a complex array of factors. Therefore, investigating the factors influencing the national carbon price is essential for enhancing the national carbon market and facilitating the transition to a green, low-carbon economy.

Existing research on the influencing factors of carbon price focuses on the European Union carbon market and the Chinese pilot carbon market. Early studies were dominated by the EU carbon market, where energy prices were generally recognised as the core influencing factor. For example [4], found that there is a significant driving effect of Brent crude oil, natural gas and coal prices on EU carbon prices, which is directly related to the high proportion of fossil fuels in the EU energy structure. However, with the establishment of China’s pilot carbon market, scholars have found significant heterogeneity in the influencing factors. Spillover analyses by Liu et al. show that Hubei carbon prices are sensitive to treasury yields, Guangdong carbon prices are dominated by crude oil prices, and Shenzhen carbon prices are highly correlated with air quality [5]. Such differences may stem from differences in pilot market design (e.g., industry coverage, quota allocation method) and regional economic structure characteristics (e.g., Guangdong’s externally oriented economy is sensitive to international energy prices). Notably, Qu et al.’s study of the Shanghai carbon price further reveals the path-dependence effect of historical prices, hinting at the non-effective characteristics of emerging carbon markets [6].

In terms of research methodology, the existing literature employs a rich variety of analytical tools, and this methodological diversity expands the research perspective. For example [7], applied the ICEEMDAN-HC method and quantile regression to analyze China’s pilot carbon markets, identifying that financial and energy markets are the primary drivers, with effects varying across time frequencies and market conditions. [8] used a nonparametric additive regression model to explore potential linear connections between carbon prices and influencing factors, discovering that coal prices and the real economy exhibit an inverted U-shaped nonlinear effect on carbon prices. [9] used a VAR-VEC model to study the interactions between energy prices, macroeconomic factors, air quality, and carbon prices in Hubei Province, China, identifying stable long-term equilibrium links between these elements. [10] employed the GA-BP-MIV model to investigate the determinants of regional carbon prices in China. Their analysis revealed that energy prices, macroeconomic conditions, industrial development, and exchange rates are critical factors influencing carbon prices. In contrast, policy measures and international carbon prices were found to have a generally limited impact on regional carbon prices. Notably, policy exhibited a modest positive effect, which was statistically significant only in Beijing and Shenzhen, while temperature demonstrated negligible influence on regional carbon prices. Overall, the diversity of available methodologies both reflects the complexity of the mechanisms influencing carbon prices and suggests the need for a more systematic assessment of the conditions of applicability and limitations of different methodologies.

To sum up, the existing research object of carbon price influencing factors mainly focuses on the EU carbon market and China’s pilot carbon market, in fact, the development of the pilot carbon market is extremely unbalanced between the pilot carbon market, so there is a big difference in the conclusions of the analysis of the pilot carbon market as the object of the research, and the same EU carbon market has a high degree of maturity and a deep degree of financialisation, and the results of the research are not applicable to the national carbon market. After the establishment of the national carbon market in China, scholars have also begun to study the national carbon market, but most of them are qualitative research, and there are fewer quantitative studies [11]. Therefore, this study takes the national carbon market as the research object to analyse the influencing factors, which enriches the research on the national carbon market and is of great significance for improving the national carbon emissions trading system. In terms of the systematicity of the influencing factors, the existing research focuses on traditional factors such as energy prices and macroeconomics, with insufficient examination of the indicators of the electric power industry, while the national carbon market, as a single market dominated by the electric power industry, is special in its price formation mechanism. Therefore, in this study, in addition to the traditional factors to consider the influence of factors, also added the electric power industry indicators, forming a new combination of variables. Regarding method selection, it is advantageous to use the VEC model for empirical analysis because it does not impose strict requirements on data stationarity compared to VAR and GARCH models, and can better reveal the relationships between variables, thereby fulfilling the research objectives of the present study. Thus, for this study, the national carbon market was taken as the research object, and daily data from July 16, 2021, to August 31, 2024 were selected, for a total of 760 sets of data after non-trading days were excluded. Energy prices, macroeconomic conditions, the development of the power industry, international carbon prices, carbon emissions from the power industry, and the trading volume of national carbon emission quotas (hereinafter referred to as trading volume) were chosen as independent variables. A VEC model was applied to investigate the dynamic interactions between the national carbon price and these variables. Furthermore, impulse response analysis and variance decomposition were conducted to identify the main factors influencing carbon price movements, offering valuable insights for mitigating market uncertainty and improving the carbon trading framework.

## I. Theoretical analysis of factors influencing carbon prices

**Fig 1:**
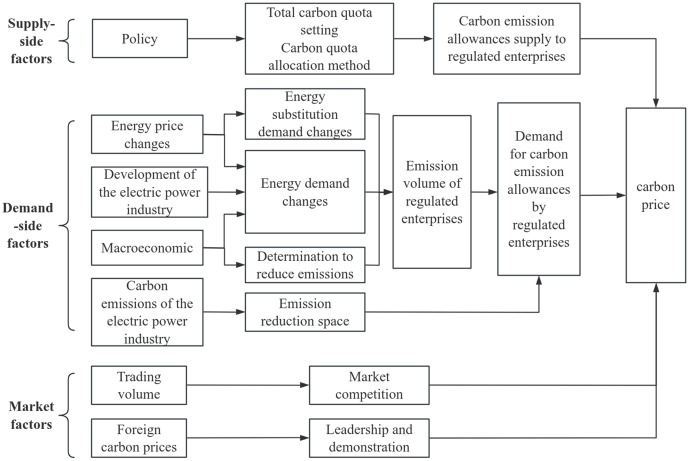
Pathway influencing carbon prices.

Carbon emission allowances are as a “commodity” created to control GHG emissions. Since the price of a commodity is mainly influenced by supply-demand relationships and the trading market, carbon price is analyzed from the perspectives of supply side, demand side, and market. The impact path of carbon prices is shown in Fig 1.

### 1.1 Supply-side analysis of carbon emission allowances

China’s national carbon market is still in its infancy, and the government plays a significant market-guiding role, with relevant government policies having an important impact on the carbon market and carbon prices. The supply of carbon emission allowances is mainly affected by policies, as reflected in the following two aspects. First, in the cap-and-trading system, the supply of carbon emission allowances is determined by the total quota set by the government and the voluntary Chinese Certified Emission Reduction (CCER) projects, with the CCER projects accounting for only a small portion and the total quota issued by the government playing a decisive role. Since production scales and the technical conditions of enterprises are fixed, a larger total quota leads to a smaller overall carbon emission gap for each enterprise and a lower carbon price; conversely, smaller total quota results in a larger overall carbon emission gap for each enterprise and a higher carbon price. Second, the methods for allocating carbon quotas among enterprises, such as free allocation, paid allocation, and the proportion of paid allocation, can also affect the demand of enterprises for carbon emission allowances, and carbon emission allowances are essentially a “commodity” created to control GHG emissions. Therefore, the carbon quota policy and its allocation mechanism influence carbon prices from the supply side of emission allowances. [12] used a logit model to study the carbon market in China, and the results showed that short-term carbon prices are significantly affected by environmental regulations and CO2 emission policies. When the government formulates the quota policy, it needs to consider the GHG emission reduction target and economic development expectation of the whole society, which means that the policies in these aspects will also affect the carbon price. However, carbon quota policy, quota allocation mechanism and related policies are all government regulatory instruments rather than market behavior, so it is also possible that the supply is difficult to respond to market changes, resulting in a carbon price that does not effectively reflect the true cost of emission reduction and a failure of the price adjustment function. Therefore, a reasonable carbon market design needs to embed market-oriented elements in government regulation to enhance the flexibility of the supply side.

### 1.2 Demand-side analysis of carbon emission allowances

The ultimate demand side of carbon emission allowances is the emission-control enterprises, and changes in the demand for carbon emission allowances by enterprises will lead to fluctuations in the carbon price, so the influencing factors of the demand for carbon emission allowances by enterprises also become the influencing factors of the carbon price. These influencing factors mainly include energy prices, the macroeconomy, the development of the power industry, and the carbon emissions of the power industry.

#### 1.2.1 Energy prices.

Coal, oil, and natural gas are the main fossil fuels that drive China’s economic development, and the combustion of fossil fuels is the main cause of the sharp increase in GHG emissions. Among the three, coal produces the most carbon emissions when burned, followed by oil, both are considered high-carbon energy [13], while natural gas has lowest carbon emissions and is classified as a clean energy. In 2023, China’s energy consumption structure will show an adjustment trend. The proportion of coal consumption will be 55.3%, a decrease of 0.7 percentage points from 2022; the proportion of oil consumption will be 18.3%, an increase of 0.3 percentage points; and the proportion of clean energy consumption will reach 26.4%, an increase of 0.4 percentage points [14]. Coal remains the main source of energy consumption.Energy prices affect carbon prices in two main ways. Firstly, changes in energy prices influence energy demand, and secondly, changes in energy prices triggers substitution effects among energy sources, influencing enterprises’ energy consumption structures. In the case of unchanged production capacity of emission-control enterprises, changes in the energy consumption structure directly affect their carbon emissions, which in turn affects their demand for carbon emission allowances; an increase in the demand for carbon emission allowances prompts a higher carbon price, while a decrease in the demand for carbon emission allowances lowers the carbon price. Rising coal prices result in reduced coal consumption [15], leading to lower CO2 emissions and thereby curbing carbon price increases. Additionally, the relationship between oil prices and carbon prices is complex. Higher oil prices could spur firms to increase coal use, pushing up carbon prices [16]; on the other hand, an increase in oil prices prompts enterprises to increase their use of clean energy, causing carbon prices to fall. [8] found that oil price has an m-shaped nonlinear effect on carbon price through a nonparametric additive regression model. The increase of natural gas price will encourage enterprises to expand the use of low-cost energy sources such as coal and oil, so the natural gas price also has an important impact on the carbon price.

#### 1.2.2 Macroeconomy.

The macroeconomy affects carbon prices in two main ways. First, by influencing the total social demand, the macroeconomy affects the production capacity and energy demand of emission-control enterprises, which further affects the demand of emission-control enterprises and leads to fluctuations in the carbon price. Chevallier utilized a Markov-switching vector autoregressive model to identify a positive correlation between macroeconomic conditions and EU carbon prices [17]. When the macroeconomic environment is robust, overall societal demand grows, enterprises’ energy consumption increases, and carbon emissions rise accordingly, pushing up the need for carbon emission allowances and elevating the cost of carbon. Conversely, in times of recession, the price of carbon decreases. Additionally, the macroeconomy influences public commitment to carbon reduction, thereby affecting carbon prices. The macroeconomic conditions shape public resolve to reduce emissions and, consequently, impact investor behavior.

#### 1.2.3 Development of the electric power industry.

The power industry is currently the only emission-control sector included in the national carbon market. Thus, the overall development of the power industry is closely related to the national carbon market. The development of the power industry has a more direct impact on the production capacity and energy demand of emission-control enterprises than does the macroeconomy. Specifically, the development of the power industry reflects its future trajectory and electricity demand, which influence corporate production planning. This, in turn, shapes industrial energy consumption and ultimately drives carbon price fluctuations through shifts in carbon allowance demand.

#### 1.2.4 Carbon emissions of the electric power industry.

The power industry was the first industry included in the national carbon market, with carbon emissions reaching 4.5 billion tons, accounting for approximately 40% of the national total [18]. Currently, the national carbon market regulates both direct emissions from the thermal power industry and indirect emissions from electricity and heat consumption, making power industry emissions the primary driver of quota demand in the carbon market. The influence of these emissions on carbon prices manifests in two key ways. First, under fixed carbon quotas, the industry’s emissions directly shape enterprises’ demand for carbon allowances, thereby affecting carbon prices. Second, power sector emissions impact policy, technology, and the energy mix. Rising emissions prompt adjustments in carbon quota policies, increasing the total quota allocation. This, in turn, encourages the adoption of low-carbon technologies and boosts clean energy usage, enhancing enterprises’ emission reduction potential and further influencing carbon prices.

### 1.3 Market analysis of carbon emission allowances

The trading of carbon emission allowances is conducted within the carbon market, making market dynamics a key driver of carbon price fluctuations. Fang et al. highlighted that carbon market volatility significantly influences carbon prices [19,20]. Findings from general equilibrium models further confirm the strong linkage between carbon prices and carbon market mechanisms [21]. Beyond market mechanisms and policies, factors such as trading volume and international carbon prices also play a critical role in shaping carbon price trends.

#### 1.3.1 Trading volume.

Under the carbon market mechanism, the government determines the total carbon emission quota at the start of a new compliance cycle and distributes it to emission-control enterprises, which are required to fulfill their quota obligations within the cycle. Variations in the trading volume of carbon emission quotas reflect both the demand for allowances among enterprises and stimulate competition within the carbon market. Given the fixed total quota, fluctuations in trading volume influence the decisions of emission-control enterprises and investors, thereby driving carbon price volatility. It is worth noting that trading activity tends to surge with the end of the compliance period, leading to a subsequent increase in carbon market prices.

#### 1.3.2 Foreign carbon prices.

Europe’s industrial development peaked earlier than that of other regions, leading to an awareness of the importance of environmental protection in advance and proactive actions on carbon emissions reduction. The EU Emissions Trading System, established in 2005, is now a relatively mature carbon market, whereas China did not establish a relatively unified national carbon market until 2021, so there is a long road ahead. The EU carbon market serves as a guide and model for China’s carbon market. Relevant Chinese scholars and experts draw on the relatively mature and stable practices of the EU carbon market to better stabilize China’s carbon market, improve its pricing mechanism, and manage risks. The carbon emission reduction tasks are jointly determined by the United Nations committee and the governments of various countries, and participating countries strictly limit China’s carbon emissions to meet reduction goals, thereby affecting carbon prices in China. Thus, foreign carbon prices affect the fluctuations in domestic carbon prices.

## II. Empirical research

### 2.1. Model introduction

#### 2.1.1 VEC model.

The Vector Autoregression (VAR) model, introduced by Christopher Sims in 1980, is a widely employed econometric model. It primarily aims to elucidate how temporal variations in a set of variables are influenced by both their own past values and those of other variables within a time series context. Here, Y_t_ represents a K-dimensional vector of endogenous variables, p denotes the lag order, A signifies the coefficient matrix to be estimated, C is a constant vector, ε_t_ is a K-dimensional vector of random disturbances, and T indicates the sample size. The VAR(p) model is expressed as follows:


$$Y_{t}=C+A_{1}Y_{t-1}+A_{2}Y_{t-2}+...+A_{p}Y_{t-p}+\varepsilon_{t},t=1,2,...,T$$

The VEC model is a constrained VAR model, which adds a cointegration constraint to the VAR model. This means that the variables can be nonstationary, but they must have a cointegration relationship. The general cointegration regression is expressed as follows:


$$\Delta Y_{t}=\alpha \beta^{\prime }Y_{t-1}+\sum_{i=1}^{p-1}\Gamma_{i}\Delta Y_{t-1}+\varepsilon_{t}$$

where α and β are both n × r matrices. α is a short-term parameter and serves as the weight of the equilibrium error correction term, reflecting the adjustment speed of the model from a nonequilibrium state to a long-term equilibrium state. β is a long-term parameter and is a matrix that consists of cointegration vectors. The error term ε_t_ (t = 1, 2, …, k) in each equation is assumed to be stationary. A cointegration system can be represented in multiple ways, with the error correction model being a frequently employed approach to address such issues.


$$\Delta Y_{t}=\alpha ecm_{t-1}+\sum_{i=1}^{p-1}\Gamma_{i}\Delta Y_{t-1}+\varepsilon_{t}$$

$$ecm_{t-1}=\beta^{\prime }Y_{t-1}$$

where ecm_t-1_ denotes the error correction term, capturing the long-term equilibrium relationship.

#### 2.1.2 Impulse response.

The VAR model captures the aggregate influence among variables, focusing solely on their static impact within the model without analyzing the specific extent of influence one variable exerts on others. However, Impulse Response Functions (IRFs) depict how endogenous variables react to an error shock. Specifically, this method involves observing the responses of other variables by applying a one-time impulse (unit shock) to a particular variable. IRF describes the impact on the current and future values of endogenous variables after applying a standard deviation-level shock to the random error term.

Suppose we consider a system containing k variables, where the i-th variable is denoted as y_i_. The VAR(p) model of this system is given as:


$$y_{t}=C+A_{1}y_{t-1}+A_{2}y_{t-2}+...+A_{p}y_{t-p}+\varepsilon_{t}$$

where c is a constant vector, A_1_, A_2_, …, A_p_ are k × k coefficient matrices representing the lagged effect of each variable, and ε_t_ is a k-dimensional error term. IRF describes the response of other variables y_i_ (i ≠ j) when the value of the j-th variable y_j_ is increased by one unit at time t = 0. IRF is expressed as follows:


$$IRF_{ji}(h)=\frac{\partial y_{j,t+h}}{\partial \varepsilon_{i,t}}$$

where IRF_ji_(h) represents the impact of the j-th variable on the i-th variable, and h denotes the number of time lag periods. IRF clearly represents the extent to which an impulse shock to the j-th variable y_j_ at the current time (t = 0) affects the i-th variable over the subsequent h periods.

### 2.2 Sample selection and data sources

The research object of this paper is the national carbon market, so starting from the time when the national carbon market started online trading, the daily data of the interval from 16 July 2021 to 31 August 2024 were selected, excluding non-trading days, and a total of 760 sets of data were obtained.

(1) National carbon price. The average daily trading price of national carbon emission allowances as the national carbon price (CEA) data (from CSMAR) is the explanatory variable, and the rest of the variables are the explanatory variables.
(2) The macroeconomy. With the continuous improvement of China’s capital market, stock indices increasingly reflect the overall economic situation [22]. Therefore, the Shanghai and Shenzhen 300 Index (CSI300) was chosen as a proxy for China’s macroeconomic development level (data obtained from CSMAR)
(3) The development of the electric power industry. The development of the electric power industry was assessed using the Shanghai and Shenzhen 300 Electric Power Index (EL300) as a key metric (data from CSMAR).
(4) Foreign carbon prices. Among global carbon markets, the EU carbon market stands out as the largest and most established. Consequently, the average daily price of EU carbon allowances (EUA) was selected to represent the foreign carbon price. (data from Wind database).
(5) Trading volume of national carbon emission quotas. The data for trading volume (VOLUME) were obtained from CSMAR.
(6) Carbon emissions from the power industry. The data on carbon emissions from the electric power industry (ELCO_2_) were obtained from Carbon Monitor. The Carbon Monitor is an international initiative that brings together ten research groups to monitor changes in CO2 emissions from fossil fuel combustion and cement production since 1 January 2019 at the national level. The Carbon Monitor covers high-resolution activity data on global emissions from sectors such as electricity, industry, ground transport, air transport, and residential consumption, covering global CO2 emissions at a daily resolution. Daily CO2 emissions are estimated from a diverse range of activity data, including: hourly to daily electrical power generation data of 29 countries, monthly production data and production indices of industry processes of 62 countries/regions, daily mobility data and mobility indices of road transportation of 416 cities worldwide.
(7) Energy prices. Oil price (OIL), coal price (COAL) and natural gas price (GAS) were selected to represent energy prices. The Daqing Oilfield is China’s largest oilfield. As of March 26, 2023, it has cumulatively produced over 2.5 billion tons of crude oil, accounting for 36% of China’s total onshore crude oil production. Thus, the crude oil price of the Daqing Oilfield was chosen as representative of China’s oil prices [23] (data sourced from Tonghuashun iFinD). In view of data availability, the coal price was represented by China’s coal price (data sourced from Tonghuashun iFinD), and the natural gas price was represented by China’s natural gas price (data sourced from ppi.cn).

The fluctuation of the trading volume is large, there are extreme values, and there are large magnitudes with other price variables, so in order to reduce the magnitude difference and make the data smoother, all the data are logarithmically processed. All empirical results are processed using Eviews12. Table 1 lists the descriptions of the variables.

**Table 1:** Descriptions of variables.

Variable symbol	Variable	Indicator	Data source
CEA	National carbon price	National carbon price	CSMAR
CSI300	Shanghai and Shenzhen 300 Index	Macroeconomy	CSMAR
EL300	Shanghai and Shenzhen 300 Electric Power Index	Development of the electric power industry	CSMAR
EUA	EU carbon price	Foreign carbon price	Wind
VOLUME	Trading volume of national carbon emission quotas	Trading volume of national carbon emission quotas	CSMAR
ELCO_2_	Carbon emissions from the power industry	Carbon emissions from the power industry	Carbon Monitor
OIL	Daqing Oilfield crude oil spot price	Energy prices	iFin
COAL	China coal price		iFin
GAS	China natural gas market price		Business agency

### 2.3 Empirical analysis

#### 2.3.1 Stationarity test.

To avoid spurious regression, a stationarity test was performed on the data. The augmented Dickey‒Fuller (ADF) test was used to test the stationarity of each variable. These variables usually do not increase or decrease over time, and thus do not have significant time trends or intercept terms. Thus, when the ADF test was conducted, no time trends or intercept terms were added. The test outcomes are presented in Table 2. The P-values for the original series of all variables exceed 0.05, indicating that the original series are non-stationary. Following first-order differencing, the results reveal that all series achieve stationarity. Consequently, all series are first-order integrated, meeting the necessary condition for the cointegration test.

**Table 2:** Results of stationarity test of variables.

Variable	ADF test value	Critical value at each significance level		P-value	Test result
		1%	5%		
LNCEA	1.089644	−2.568041	−1.941245	0.9286	Nonstationary
LNVOLUME	−1.391668	−2.568033	−1.941244	0.1527	Nonstationary
LNCSI300	−1.527569	−2.568012	−1.941241	0.1189	Nonstationary
LNEL300	1.292967	−2.568012	−1.941241	0.9507	Nonstationary
LNEUA	0.241138	−2.568012	−1.941241	0.756	Nonstationary
LNCOAL	−0.258247	−2.568024	−1.941242	0.593	Nonstationary
LNOIL	0.171662	−2.568012	−1.941241	0.7358	Nonstationary
LNGAS	0.027669	−2.568016	−1.941241	0.6913	Nonstationary
LNCO_2_	−0.035122	−2.568012	−1.941241	0.6708	Nonstationary
DLNCEA	−15.92806	−2.568041	−1.941245	0	Stationary
DLNVOLUME	−19.66936	−2.568033	−1.941244	0	Stationary
DLNCSI300	−27.5538	−2.568016	−1.941241	0	Stationary
DLNEL300	−26.87936	−2.568016	−1.941241	0	Stationary
DLNEUA	−29.0391	−2.568016	−1.941241	0	Stationary
DLNCOAL	−10.84602	−2.568024	−1.941242	0	Stationary
DL NOIL	−29.30218	−2.568016	−1.941241	0	Stationary
DLNGAS	−19.06157	−2.568016	−1.941241	0	Stationary
DLNCO_2_	−28.71251	−2.568016	−1.941241	0	Stationary

#### 2.3.2 Determination of the optimal lag order.

To establish a suitable VAR or VEC model, determining the optimal lag order is essential. In this research, the optimal lag order was identified using the LR, AIC, SC, HQ, and FPE criterion. As shown in the results in Table 3, the optimal lag order selected is 1 by SC, 2 by HQ, and 3 by both AIC and FPE. Thus, the lag order of 3, chosen by more criteria, was selected as the optimal lag order.

**Table 3:** Results of the optimal lag order.

Lag	LogL	LR	FPE	AIC	SC	HQ
1	12545.01	NA	2.72e-26	−33.32624	−32.82623*	−33.13355
2	12737.97	376.6201	2.02e-26	−33.62558	−32.62556	−33.24020*
3	12832.79	182.8051	1.94e-26*	−33.66255*	−32.16251	−33.08448
4	12900.45	128.8004	2.01e-26	−33.62687	−31.62682	−32.85611
5	12966.79	124.6952	2.10e-26	−33.58767	−31.08760	−32.62422
6	13026.13	110.1104	2.22e-26	−33.52975	−30.52967	−32.37361
7	13084.60	107.0973*	2.36e-26	−33.46952	−29.96943	−32.12069
8	13141.52	102.8894	2.53e-26	−33.40515	−29.40504	−31.86363
9	13186.03	79.37564	2.79e-26	−33.30757	−28.80746	−31.57337
10	13222.21	63.64206	3.16e-26	−33.18772	−28.18759	−31.26082
11	13274.20	90.22432	3.42e-26	−33.11016	−27.61002	−30.99057
12	13331.32	97.74944	3.66e-26	−33.04631	−27.04616	−30.73404

#### 2.3.3 Cointegration test.

The cointegration test assesses whether a long-term stable equilibrium exists among variables. Even if individual variables are nonstationary, identifying such an equilibrium allows for the establishment of a VEC model. Common cointegration tests include the Engle-Granger test and the Johansen test. Due to its effectiveness in analyzing multiple variables, the Johansen test was utilized in this study. The results, displayed in Tables 4 and 5, show that under the null hypothesis of no cointegration, the trace statistic is 231.0067, exceeding the critical value of 197.3709 at the 5% significance level, with a P-value below 0.05. Consequently, the null hypothesis is rejected, indicating the presence of at least one cointegration relationship. Under the null hypothesis of at most one cointegration relationship, the trace statistic of 140.8083 is less than the critical value of 159.5297, leading to the acceptance of the null hypothesis and suggesting the existence of at most one cointegration relationship. The maximum eigenvalue test yields consistent results, confirming one cointegration relationship, which signifies a long-term equilibrium between the national carbon price and the variables.

**Table 4:** Results of the Johansen cointegration test(a).

Hypothesis	Eigenvalue	Trace statistic	Critical value at the 5% significance level	P-value
None *	0.112327	231.0067	197.3709	0.0003
At most 1	0.056369	140.8083	159.5297	0.3266
At most 2	0.039812	96.88727	125.6154	0.6971
At most 3	0.033038	66.13331	95.75366	0.8372
At most 4	0.021484	40.70069	69.81889	0.9363
At most 5	0.015390	24.26009	47.85613	0.9368
At most 6	0.010460	12.51942	29.79707	0.9123
At most 7	0.005439	4.559693	15.49471	0.8536
At most 8	0.000570	0.431468	3.841465	0.5113

**Table 5:** Results of the Johansen cointegration test(b).

Hypothesis	Eigenvalue	Largest eigenvalue root	Critical value at the 5% significance level	P-value
None *	0.112327	90.19834	58.43354	0.0000
At most 1	0.056369	43.92103	52.36261	0.2794
At most 2	0.039812	30.75396	46.23142	0.7360
At most 3	0.033038	25.43262	40.07757	0.7400
At most 4	0.021484	16.44060	33.87687	0.9413
At most 5	0.015390	11.74066	27.58434	0.9426
At most 6	0.010460	7.959731	21.13162	0.9061
At most 7	0.005439	4.128225	14.26460	0.8456
At most 8	0.000570	0.431468	3.841465	0.5113

The normalized cointegration equation is as follows:


$$\begin{array}{c} LNCEA=-1.448LNCOAL+0.383LNCSI300+1.195LNEL300-0.757LNELCO2\\ -0.368LNEUA+0.336LNGAS+0.465LNOIL+0.063LNVOLUME \end{array}$$

From the cointegration equation, it can be seen that under the long-term equilibrium relationship, when other variables are constant, a 1% increase in coal price decreases the national carbon price by 1.448%; when other variables are constant, a 1% increase in Power 300 index increases the national carbon price by 1.195%; when other variables are constant, a 1% increase in power industry carbon emissions increase by 1%, the national carbon price decreases by 0.757%; when other variables are held constant, a 1% increase in the EU carbon price decreases the national carbon price by 0.368%; when other variables are held constant, a 1% increase in the price of natural gas increases the national carbon price by 0.336%; when other variables are held constant, a 1% increase in the price of oil increases the national carbon price by 0.465; and when other variables are held constant, a 1% increase in turnover increases the national carbon price increases by 0.063%. The negative elasticity coefficient of EU carbon prices may reflect the policy competition effect under the fragmentation of global carbon markets: When the EU strengthened emission reduction by raising the carbon price, the national carbon market was still in the early stage of establishment, following the strategy of seeking progress while maintaining stability, and the institutional system was still in the process of continuous improvement, so the level of the carbon price was still maintained at a relatively low state in order to achieve a balance between environmental protection and industrial development. This dynamic relationship may change as the national carbon market matures and international climate policy coordination deepens. The elasticity coefficient of carbon emissions from the power industry is negative, which may be due to the fact that in the long-term process, the increase in carbon emissions from the power industry has prompted enterprises to enhance their low-carbon emission reduction technology level and increase the space for carbon emission reduction, and the demand for carbon emission rights from enterprises has decreased, while the government has also increased the supply of carbon emission rights, which has led to the reduction of carbon price for enterprises. The standard errors and t-statistics of the coefficients of the cointegration equation and VEC model are shown in Table 6.

**Table 6:** Normalized cointegration equation and VEC model.

Variables	Coefficients	Standard errors	T-statistics
LNCOAL	−1.448***	(0.28597)	[5.06231]
LNCSI300	0.383	(0.29584)	[-1.29334]
LNEL300	1.195***	(0.28126)	[-4.24910]
LNELCO2	−0.757***	(0.22112)	[3.42421]
LNEUA	−0.368**	(0.16185)	[2.27530]
LNGAS	0.336**	(0.14809)	[-2.27218]
LNOIL	0.465**	(0.20892)	[-2.22424]
LNVOLIME	0.063***	(0.00700)	[-9.04878]
CointEq1	−0.046***	(0.01089)	[-4.22090]

#### 2.3.4 Establishment of the VEC model.

The cointegration test only describes whether there is a long-term equilibrium between the variables and cannot reflect the short-term adjustment between the variables, so in order to further explore the relationship between long-term equilibrium and short-term adjustment, a VEC model is established. The estimation formula of D(LNCEA) in the VEC model is as follows:


$$D\left(LNCEA\right)=-0.046\left[\begin{array}{c} LNCEA\left(-1\right)+1.448LNCOAL\left(-1\right)-0.3828LNCSI300\left(-1\right)\\ -1.1958LNEL300\left(-1\right)+0.7578LNELCO2\left(-1\right)+0.3688LNEUA\left(-1\right)\\ -0.3368LNGAS\left(-1\right)-0.465LNOIL\left(-1\right)-0.063LNVOLUME\left(-1\right)+0.143 \end{array}\right]+0.0012$$

The negative adjustment coefficient aligns with the corrective mechanism of the VEC model. The absolute value of the adjustment coefficient is 0.046, which indicates that when deviating from the equilibrium state, there will be an adjustment strength of 0.046 to pull the national carbon price back to equilibrium. The adjustment coefficient is small because the national carbon market was established not long ago, and the relevant mechanisms remain incomplete, and unable to quickly and effectively transmit information or price fluctuations from other markets.

**Fig 2:**
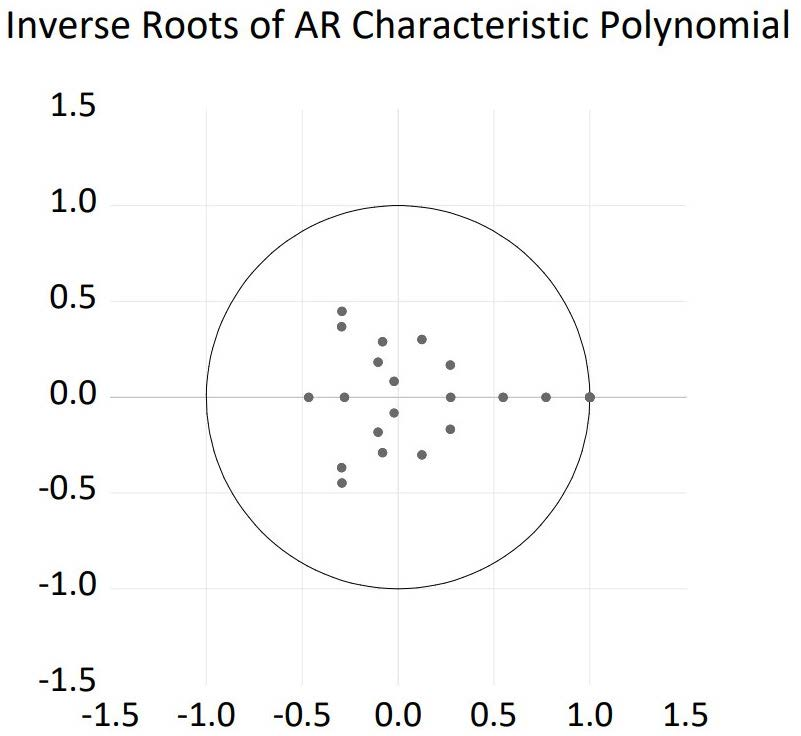
AR root test results of the VEC model.

A stability test of the VEC model was performed to examine its validity. The results in Fig 2 show that all points (positions of the unit roots) are inside the circle, indicating that the model is stable.

#### 2.3.5 Impulse response.

**Fig 3:**
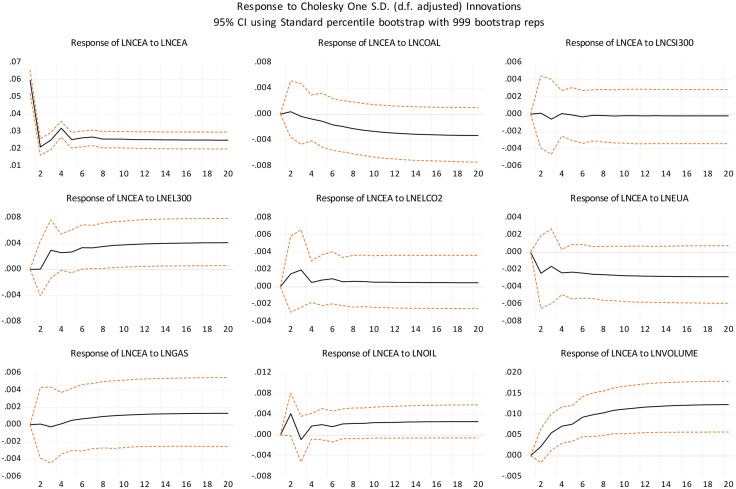
Impulse response results.

The impulse response refers to the process by which the dependent variable, namely the national carbon price, is affected when a standard deviation shock is applied to the explanatory variables, including the duration of action and the impact path. A total of 20 periods was selected, and the data were all daily data. Thus, 20 periods represent 20 days. The short-term impact was studied, and the results are shown in Fig 3, in which the horizontal axis represents the lag period, and the vertical axis indicates the effect of the shock on the national carbon price.

(1) National carbon price. The findings indicate that the national carbon price is positively impacted by its own historical price levels, with the greatest impact level occurring in the first period, starting to fluctuate after the second period, and stabilizing around the 12th period.
(2) Trading volume. Trading volume positively impacts the national carbon price, with a long lag effect, starting at zero in the first period and gradually increasing until stabilizing after the 18th period. The rise in trading volume intensifies competition within the carbon market, thereby driving up the national carbon price.
(3) Carbon emissions from the power industry. The carbon emissions from the power industry have a short-term positive effect on the national carbon price, starting at zero in the first period, gradually increasing, then decreasing after the third period, and stabilizing from the 15th period onward. This is contrary to the result under the long-term situation in the cointegration equation. The primary reason for this discrepancy lies in the short-term dynamics: rising carbon emissions from the power industry directly indicate heightened emissions by enterprises, thereby increasing their need for carbon allowances and pushing the national carbon price upward. Thus, power industry emissions have a short-term positive effect on the national carbon price. Conversely, in the long term, increased emissions from the power sector prompt policy adjustments, such as higher total carbon quotas. This also spurs enterprises to adopt low-carbon technologies and increase clean energy consumption, thereby expanding their potential for emission reduction and ultimately lowering the national carbon price. This long-term effect is opposite to the short-term direct impact of power industry emissions on the national carbon price.
(4) EU carbon price. EU carbon price negatively impacts the national carbon price, beginning with no impact in the initial period, gradually rising in the second period, showing slight fluctuations between the third and fourth periods, and stabilizing from the 16th period onward. The negative impact of EU carbon prices may stem from policy competition in global carbon markets. When the EU strengthened emission reduction by raising the carbon price, the national carbon market was still in the early stage of establishment, following the strategy of seeking progress while maintaining stability, and the institutional system was still in the process of continuous improvement, so the level of the carbon price was still maintained at a relatively low state in order to achieve a balance between environmental protection and industrial development.
(5) Energy prices. Among energy price indicators, coal price negatively impacts the national carbon price (consistent with the results of [24]), starting at zero in the first period, showing positive growth in the second period but with nearly no impact, and beginning to show negative growth after the third period. An increase in coal prices prompts enterprises to switch to alternative energy sources. Given coal’s high carbon emissions, such substitution reduces overall emissions, thereby lowering the national carbon price. Conversely, oil prices positively influence the national carbon price (consistent with the results of [25,26]). Specifically, oil price fluctuations show a brief negative impact in the third period but stabilize and turn positive from the fourth period onward. The impact of the natural gas price on the national carbon price is roughly similar to that of the oil price, but the positive impact of natural gas is slightly smaller. When the oil price increases, the rigid demand of enterprises for oil leads to an increase in the cost of reducing carbon emissions for the enterprise, which is passed on to the carbon price, resulting in a higher carbon price.. When natural gas prices rise, substituting with other energy sources results in higher carbon emissions compared to natural gas, thereby increasing the expense of emission reduction. In contrast, the continued use of natural gas has a long-term price advantage and aligns with the low-carbon policy direction.
(6) CSI 300 Index. The CSI 300 Index positively impacts the national carbon price, but its influence is small, almost approaching zero.
(7) The EL 300 Index positively impacts the national carbon price, with a significant influence that starts at zero in the first period and gradually increases until it stabilizes around the tenth period. The power industry is more tightly connected to the carbon trading market compared to the macroeconomy. As a result, its development has a more pronounced effect on the national carbon price. The positive growth in both factors enhances corporate production capacity, driving up carbon emissions and subsequently the national carbon price.

#### 2.3.6 Variance decomposition.

Variance decomposition and impulse response analysis offer complementary perspectives on the same phenomenon. Impulse response analysis measures the reaction of an economic variable to impulses from another variable, whereas variance decomposition quantifies the contribution of each structural shock to the variance of other variables, highlighting the differences in the magnitude of their impacts. To ensure consistency in the research, the number of periods analyzed is set to 20, matching the impulse response analysis. The variance decomposition results for each variable’s impact on the carbon price are shown in Table 7. The findings reveal that the variance of the prediction error of the national carbon price is predominantly influenced by its own historical price fluctuations. Although this influence diminishes over time, it still accounts for more than 85% of the explanatory power. The impact of trading volume on the national carbon price starts at zero in the first period, gradually increases, and reaches an explanatory level of 11% by the 20th period. The effects of coal price, EL 300 Index, EU carbon price, natural gas price, and crude oil price on the national carbon price are initially zero but grow slightly over time, though their contributions remain minimal. The CSI 300 Index’s impact on the national carbon price starts at zero, fluctuates in the subsequent periods, and eventually stabilizes with a negligible contribution, almost zero. Likewise, the impact of carbon emissions from the power industry begins at zero, increases gradually, starts to decline in the sixth period, and stabilizes around the 15th period.

**Table 7:** Variance decomposition results.

Period	LNCEA	LNCOAL	LNCSI300	LNEL300	LNELCO_2_	LNEUA	LNGAS	LNOIL	LNVOLUME
1	100.0000	0.000000	0.000000	0.000000	0.000000	0.000000	0.000000	0.000000	0.000000
2	99.24686	0.003344	0.000353	2.97E-05	0.054796	0.148444	0.000137	0.420285	0.125750
3	98.35705	0.005459	0.007324	0.185564	0.126602	0.184792	0.001526	0.377132	0.754553
4	97.50675	0.014229	0.006026	0.264602	0.107040	0.249932	0.001413	0.358139	1.491865
5	96.60363	0.030926	0.005523	0.345993	0.104138	0.304932	0.005165	0.380700	2.218989
6	95.46522	0.064044	0.006225	0.462103	0.104379	0.352312	0.010577	0.374702	3.160439
7	94.40328	0.102375	0.005821	0.550642	0.097565	0.397091	0.017440	0.392240	4.033545
8	93.36227	0.149504	0.005638	0.642012	0.093161	0.438740	0.025940	0.412850	4.869881
9	92.34098	0.200050	0.005622	0.729646	0.089183	0.474971	0.034586	0.430092	5.694865
10	91.39630	0.251812	0.005493	0.808286	0.084897	0.508585	0.043253	0.450324	6.451054
11	90.51376	0.303676	0.005416	0.882216	0.081273	0.538781	0.051842	0.469789	7.153249
12	89.69403	0.354067	0.005367	0.950158	0.077956	0.565953	0.060023	0.487919	7.804528
13	88.94156	0.402258	0.005309	1.012109	0.074884	0.590647	0.067762	0.505372	8.400095
14	88.24922	0.447953	0.005265	1.069051	0.072132	0.612987	0.075040	0.521641	8.946714
15	87.61333	0.490855	0.005229	1.121134	0.069624	0.633254	0.081819	0.536725	9.448027
16	87.02999	0.530954	0.005195	1.168805	0.067335	0.651710	0.088119	0.550759	9.907128
17	86.49406	0.568318	0.005167	1.212540	0.065251	0.668535	0.093963	0.563740	10.32843
18	86.00126	0.603054	0.005141	1.252689	0.063344	0.683918	0.099377	0.575744	10.71547
19	85.54755	0.635324	0.005118	1.289613	0.061595	0.698022	0.104392	0.586856	11.07153
20	85.12905	0.665297	0.005097	1.323643	0.059989	0.710983	0.109040	0.597142	11.39976

During the 18th period, the contributions of various factors to the carbon price fluctuations stabilize. We conducted analysis and research based on the stabilized contribution rate of each factor. In the 18th period, various influencing factors in the national carbon market are ranked by contribution as follows: own historical prices > trading volume > EL 300 Index > EU carbon price > coal price > oil price > natural gas price > carbon emissions from the power industry > CSI 300 Index. The carbon emissions of power industry had a limited impact on the national carbon price, mainly because of their limited ability to rapidly influence the wider carbon market in the short term. More specifically, the emissions from regulated enterprises directly affect their demand for carbon allowances, while the broader industry’s impact on the carbon market exhibits a significant lag. Additionally, the CSI 300 Index has an insignificant effect on the national carbon price, as the power industry’s development more directly impacts the carbon market than the macroeconomy does. These variance decomposition findings align with the impulse response results, further validating the effectiveness of the VEC model.

## III. Conclusions and recommendations

### 3.1 Conclusions

In this study, the national carbon price was chosen as the dependent variable, while coal price, oil price, natural gas price, CSI 300 Index, EL 300 Index, EU carbon price, power sector emissions, and trading volume were used as independent variables. The VEC model was applied to examine the long-term equilibrium relationships between these variables and the adjustment process from short-term deviations to long-term equilibrium. Furthermore, impulse response analysis and variance decomposition were performed to assess the short-term dynamic impacts of each variable on the national carbon price. The main findings are outlined below:

(1) A long-term equilibrium relationship is observed between the national carbon price and each variable. When the carbon price deviates from the equilibrium level, it will gradually converge to the long-term equilibrium value. The absolute value of the adjustment coefficient is 0.046, indicating a weak long-term adjustment and a prolonged smoothing cycle. This suggests that the national carbon price takes significant time to revert to equilibrium after fluctuations, largely due to the imperfect trading system of the national carbon market and delays in information disclosure and transmission.
(2) The fluctuations in national carbon price are affected mainly by its own historical prices, with a contribution rate of 86.001. Trading volume positively impacts the national carbon price, ranking second in contribution with a rate of 10.715, but its impact on the national carbon price has a strong lag effect. The EL 300 Index ranks third in contribution to the national carbon price fluctuations, with a rate of 1.253. The stronger the performance of the power industry, the higher the national carbon price. the large influence of the power industry underscores its unique position as the sole sector included in the national carbon market, demonstrating a strong correlation with the national carbon price, consistent with reality.
(3) Among energy price indicators, coal price exerts the most significant influence on the national carbon price, reflecting China’s coal-dominated energy consumption structure. Coal price negatively affects the national carbon price, whereas oil and natural gas prices have a positive impact. The ranking of energy prices’ influence on the national carbon price is as follows: coal price > oil price > natural gas price.
(4) Carbon emissions from the power industry have a positive impact on the national carbon price in the short term and a negative impact in the long term. In the short term, an increase in carbon emissions from the power industry directly reflects an increase in corporate carbon emissions, leading to higher demand of enterprises for carbon emission allowances and thus a rise in the carbon price. However,in the long term, an increase in carbon emissions from the power industry causes changes in the carbon quota policy and an increase in the total quota. This promotes the development of low-carbon emission reduction technologies for enterprises and causes an increase in the proportion of clean energy consumption, so enterprises’ potential for carbon emission reduction increases, resulting in a decrease in the carbon price. This results in a direction opposite to the direct impact of carbon emissions from the power sector on the carbon price in the short term. The CSI 300 index has a positive impact on the national carbon price, but to a lesser extent. The EU carbon price has a negative impact on the national carbon price, probably because under the policy competition in the global carbon market, when the EU strengthened emission reduction by raising the carbon price, the national carbon market was still in the early stage of establishment, following the strategy of seeking progress while maintaining stability, and the institutional system was still in the process of continuous improvement, so the level of the carbon price was still maintained at a relatively low state in order to achieve a balance between environmental protection and industrial development.

In general, fluctuations in the national carbon price remain largely self-driven, with other market factors having an impact on the national carbon price but to a lesser extent. This indicates that the national carbon market was established not long ago and that its market effect is not yet prominent. Nevertheless, the gradually increasing degree of influence over time suggests that this market effect is gradually strengthening. With the expansion of the national carbon market involving more industries and the introduction of diversified participants, the factors affecting the carbon price will become more complex; when the market matures, the fluctuation of the carbon price will reflect the fundamentals of the market supply and demand, the price discovery function will tend to be perfected, and the market effect will be significantly enhanced, which will ultimately lead to the formation of a more market-oriented and more efficient carbon pricing mechanism.

### 3.2 Recommendations

Based on the above conclusions and taking into account the actual situation of the national carbon market, the following recommendations are put forward to improve the institutional system of the national carbon market.

(1) **Improving the pricing mechanism.** As the market effect of the current national carbon market is not obvious, the market regulation mechanism is weak. And considering that all carbon allowances in the current national carbon market are allocated free of charge, a compensated allocation mechanism has not yet been introduced. To encourage enterprises to engage in technological innovation and promote energy transformation, the national carbon market should appropriately reduce free quotas, increase the use of paid allocations for carbon emission allowances, and gradually expand the proportion of paid allocations. Additionally, a quota reserve mechanism should be established to stabilize prices.
(2) **Stabilizing energy prices and optimizing the energy consumption structure.** High and low energy prices have an impact on the energy consumption preferences of enterprises, thus affecting the structure of energy consumption. Therefore, the Government should rationally control energy prices to make them more market-oriented and effectively reflect energy supply and demand. The release of advanced production capacity should be vigorously promoted, energy production capabilities enhanced, and the key role of energy enterprises in ensuring supply leveraged to increase effective supply in the energy market. Moreover, the pricing mechanism of the energy market should be innovated and improved, the market-oriented reform of energy prices should be deepened, and energy prices should be stabilized. Additionally, it should improve the structure of energy consumption, encourage the adoption of renewable energy, and support internal restructuring and low-carbon transformation of enterprises.
(3) **Promoting the development of carbon financial products.** At present, the national carbon market is characterized by a single trading variety and insufficient market liquidity, making it difficult to form an effective price discovery mechanism. In the context of frequent compliance tides and large carbon price fluctuations in the carbon market, carbon financial products such as carbon futures must be actively explored and developed. Financial instruments such as carbon futures can reflect future market carbon price expectations based on policy assessments and market trends, and eventually transmit these expectations to the spot market to form a price discovery mechanism. Introducing carbon futures and options alongside spot trading in the national carbon market can attract more investment, reduce compliance costs and risks for enterprises, improve their carbon asset management, increase market activity, and enhance liquidity.
(4) **Improving the institutional system of the carbon trading market.** As the national carbon market is currently in its initial stage, the government should fully leverage its market guidance role, expedite the enhancement of the carbon market’s institutional framework, and establish comprehensive systems and mechanisms for the national carbon market. These include mechanisms for total volume control, quota allocation, trading systems, certified emission reduction management, regulatory oversight, and risk control. Furthermore, detailed rules and regulatory standards for the participation of non-compliance institutional and individual investors should be introduced to diversify trading entities and boost market activity. In particular, the involvement of financial institutions in national carbon emission trading is expected to increase market liquidity and address the current challenges such as low liquidity and high trading concentration.

### 3.3 Deficiencies of the study

This study mainly explores the influencing factors of the national carbon price from the perspective of the market, and only analyses the policy theoretically, but fails to quantify the policy factors and incorporate them into the empirical model. Future research can start from the quantification of policy impacts and the interaction mechanism between policy and market to understand the role of policy factors in carbon pricing more comprehensively.
